# Fabrication and characterization of biodegradable Zn-Ni spinel ferrite/ *β*-TCP composite ceramics exhibiting enhanced cell colonization

**DOI:** 10.1007/s10856-026-07004-7

**Published:** 2026-01-29

**Authors:** Piyapong Pankaew, Poomirat Nawarat, Jaroenporn Chokboribal

**Affiliations:** 1 Division of Industrial Materials Science, Rajamangala University of Technology Phra Nakhon, Bangkok, Thailand; 2 Materials Science Program, Phranakhon Rajabhat University, Bangkok, Thailand

## Abstract

Via a solid-state reaction route, magnetic composites of chicken eggshell-derived *β*-tricalcium phosphate (*β*-TCP, referred to as TCP in the composite system) and zinc-nickel spinel ferrite (ZNF; Zn_*x*_Ni_1‒*x*_Fe_2_O_4_, *x* = 0.2, 0.4, 0.6, or 0.8) were successfully fabricated. Discs were prepared by uniaxial pressing of milled ZNF/TCP powders and sintered at 1200 °C. Cytocompatibility of all composites was confirmed by SEM observations of human osteoblasts (h-OBs) and MTT assays. At 4-wt% ZNF addition, the composites containing Zn_0.8_Ni_0.2_Fe_2_O_4_ (Z8NF) exhibited the greatest extent of early cell spreading and were selected for further investigation. For Z8NF/TCP composites containing 4–12 wt% Z8NF, the 8–12 wt% samples demonstrated the highest levels of cell colonization, while MTT assays suggested non-cytotoxic behavior, with cell viabilities comparable to *β*-TCP. High-temperature sintering induced partial transformation of *β*-TCP to *β*-calcium pyrophosphate (*β*-CPP), as evidenced by XRD and Rietveld refinement. Increasing Z8NF content promoted *β*-CPP formation and increased composite porosity, whereas densification and Vickers hardness decreased accordingly. Rietveld refinement further indicated that the detectable crystalline Z8NF phase persisted as a minor yet stable secondary phase ( < 2 wt%) and did not participate in Ca–P lattice substitution. For the 8–12 wt% composites, saturation magnetization decreased with increasing Z8NF because of higher porosity and dilution by the non-magnetic *β*-TCP/*β*-CPP matrix, while coercivity increased owing to enhanced effective magnetic anisotropy in the more porous microstructure. Overall, the Z8NF/TCP composites combined biodegradability, bioactivity, and tunable soft-magnetic properties, suggesting their potential for bone repair and bone tissue engineering applications.

**Graphical Abstract:**

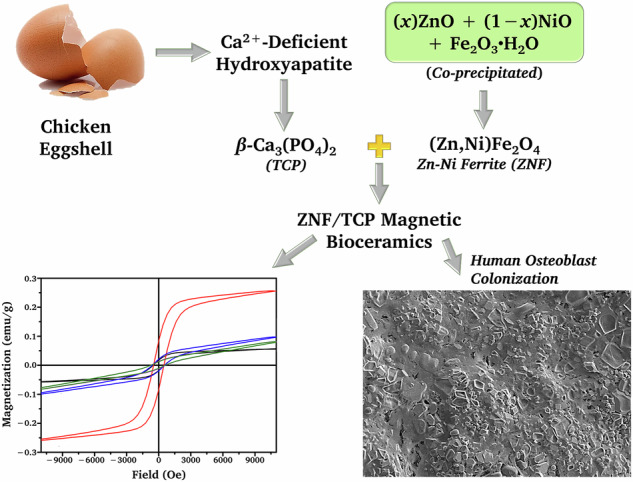

## Introduction

For biomedical applications, the two most promising calcium phosphates (CPs) are hydroxyapatite (Ca_10_(PO_4_)_6_(OH)_2_, HAp) and *β*-tricalcium phosphate (*β*-Ca_3_(PO_4_)_2_, *β*-TCP) because of their superior biocompatibility and bioactivity compared with other calcium phosphates [1]. Biodegradable materials have been increasingly investigated for bone reconstruction owing to their ability to enhance cell growth and degrade into non-toxic products that can be resorbed by the human body [2]. *β*-TCP exhibits both a higher biodegradation rate and solubility than HAp [3] and has therefore been studied in more detail and developed as a biodegradable material for bone repair and regeneration.

Currently, magnetic therapy is regarded as a novel approach for managing bone-related disorders and promoting bone healing. As a result, many studies have recently been conducted on composites of CPs with good biocompatibility and unique magnetic materials. Composites of HAp and ferrite particles, a family of iron oxide-containing magnetic ceramic such as ZnFe_2_O_4_, CoFe_2_O_4_, MgFe_2_O_4_ or Fe_3_O_4_ (magnetite) have been developed [4–7].

Seyfoori et al.[6] reported that ZnFe_2_O_4_–HAp nanoparticles supported cell viability and exhibited no adverse cytotoxic effects in MTT assays, while Sinusaite et al. [8] demonstrated through in vivo testing that Fe/Zn co-doped *β*-TCP powders were also biologically safe. Together, these findings highlight the emerging interest in ferrite–calcium phosphate composites as biologically compatible systems.

Nickel-containing compounds at controlled concentrations have been reported to exhibit acceptable biocompatibility with bone and skin tissues. Ni substitution in HAp matrix influenced cell survival, proliferation and osteogenic differentiation of MG-63 cells [9]. We previously reported the preparation of HAp and ZnFe_2_O_4_ composite ceramics via a solid-state method, which offers superior controllability of their structural and magnetic properties over other methods [10]. Despite the growing interest in bone tissue engineering, magnetic composite scaffolds and substrates remain underexplored compared to other composite forms, highlighting a promising direction for future research. There has been no investigation of composites of Zn-Ni ferrite (ZNF, (Zn,Ni)Fe_2_O_4_) and *β*-TCP to the best of our knowledge.

Numerous studies clarify that the biological response of doped spinel ferrites is governed by ion-specific and site-specific effects within the spinel lattice. Zn^2+^, which preferentially occupies tetrahedral sites, functions as an osteogenic cofactor that enhances osteoblast proliferation, alkaline phosphatase activity, and matrix mineralization, thereby improving cytocompatibility in a dose-dependent manner [11–13]. Fe^3+^, in contrast, is preferentially stabilized in octahedral sites and exhibits negligible ionic release, supporting favorable cytocompatibility while contributing primarily to magnetic functionality [14]. Ni^2+^ substitution further modulates surface charge and magnetic response, and although excessive Ni levels may induce ROS-mediated cytotoxicity, low-Ni Zn–Ni ferrites have consistently shown safe cytocompatibility and favorable cell–material interactions [10, 15]. Collectively, these mechanistic insights highlight that the relative Zn and Ni dopant contents play a critical role in determining both the biocompatibility and magnetic performance of spinel ferrite-based biomaterials.

Therefore, in this study, a promising biodegradable composite ceramic consisting of *β*-TCP (hereafter referred to as TCP in the composite system) and ZNF was proposed and fabricated. The intended application of the ZNF/TCP composites is as a bone reinforcement material, combining bioactivity with magnetically responsive properties. Lee et al. [16] reported higher biocompatibility for HAp powders prepared using calcium derived from chicken eggshells than that of HAp prepared from calcium of inorganic origin. *β*-TCP powders were prepared using chicken eggshells as a calcium source in this study.

**Fig. 1:**
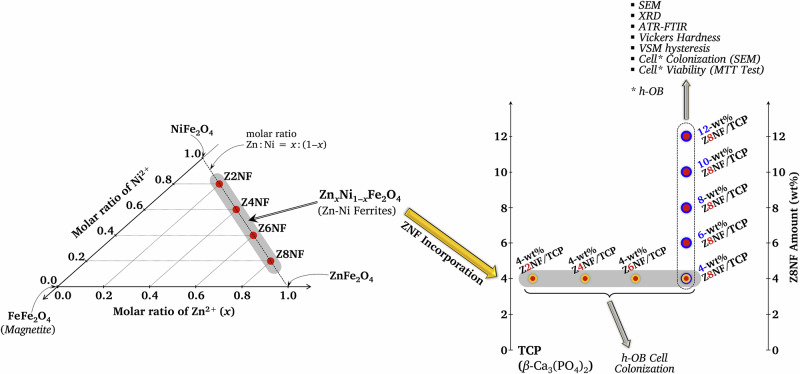
Schematic summary of the methodology of the present study

ZNF was expected to exhibit magnetic behavior as well as potentially support bone-related biological responses for future implant applications. The effects of (1) varying Zn:Ni molar ratios in the ZNF magnetic powders and (2) the ZNF content in the ZNF/TCP composites on their physical characteristics and cytocompatibility were investigated. The fabricated specimens were analyzed through various techniques, including X-ray powder diffraction (XRD), Fourier-transform infrared (FTIR) spectroscopy, scanning electron microscopy (SEM), porosity evaluation, vibrating sample magnetometry (VSM), and the Vickers hardness testing method. Direct-contact cytocompatibility of the ZNF/TCP composites with human osteoblasts (h-OBs) was assessed by cell responses on the composite surfaces (SEM) and cell viability (MTT assay) after 7 days of culture. A schematic summary of the experimental approach is given in Fig. 1.

## Experimental

### Materials

Chicken eggshells were purchased from Charoen Pokphand Foods (CPF) PCL (Bangkok, Thailand). All chemical reagents employed as raw materials were of analytical grade.

### Preparation of *β*-TCP

The membranes lining the eggshells were first removed. The collected eggshells were then rinsed thoroughly with deionized water and subsequently oven-dried at 60 °C for 24 h. The eggshells were finely ground and used as the CaO source to synthesize calcium-deficient hydroxyapatite.

CaO powder (eggshells subjected to calcination at 1100 °C for 4 h) was titrated using a 65% HNO_3_ solution. A Ca(NO_3_)_2_ solution (0.9 M) was obtained after adjusting the pH to 9 using a 30% NH_4_OH solution. A (NH_4_)_3_PO_4_ solution (0.6 M) was prepared and its pH was adjusted to 9 using a 30% NH_4_OH solution. The phosphate solution was added dropwise to the vigorously stirred Ca(NO_3_)_2_ solution over 2 h. The solutions were mixed at a Ca/P molar ratio of 1.5. The solution mixture was heated to 100 °C for 30 min and then aged for 8 h at ambient temperature. The Ca_9_(HPO_4_)(PO_4_)_5_(OH) precipitate was filtered and repeatedly rinsed with deionized water to remove residual ions. The dried precipitate (80 °C for 24 h in an oven) was ground using an agate mortar to obtain a fine calcium-deficient hydroxyapatite powder. The powder was then calcined at 700 °C for 3 h, resulting in the formation of *β*-Ca_3_(PO_4_)_2_.

### Preparations of Zn-Ni ferrites

To prepare Zn_0.8_Ni_0.2_Fe_2_O_4_ (Z8NF), 1 M aqueous solutions of Fe(NO_3_)_3_•9H_2_O (0.2 mol, 80.800 g, 200 mL), Zn(CH_3_COO)_2_•2H_2_O (0.08 mol, 17.561 g, 80 mL), and Ni(NO_3_)_2_•6H_2_O (0.02 mol, 5.816 g, 20 mL) were prepared as stock solutions and then well mixed. A NaOH solution (2 M) was heated to 85 °C. The preheated NaOH solution was added dropwise to the mixed solution over 1 h at a constant temperature of 85 °C under continuous stirring to reach the desired pH of 9. The resulting precipitates were filtered and dried at 80 °C in an oven for 24 h. The dried precipitates were then ground and calcined at 800 °C for 2 h. Zn_0.6_Ni_0.4_Fe_2_O_4_ (Z6NF), Zn_0.4_Ni_0.6_Fe_2_O_4_ (Z4NF), and Zn_0.2_Ni_0.8_Fe_2_O_4_ (Z2NF) were prepared similarly using Zn(CH_3_COO)_2_•2H_2_O, Ni(NO_3_)_2_•6H_2_O and Fe(NO_3_)_3_•9H_2_O at the molar ratios of 3:2:10, 2:3:10 and 1:4:10, respectively.

### Preparations of ZNF/TCP composites

Powders of each ZNF formulation were mixed with *β*-TCP powder using a stainless-steel vial loaded with steel grinding media and processed in a SPEX 8000-D mixer/mill (SPEX CertiPrep) for 10 min. Compaction using a uniaxial die press (ENERPAC RC104) and subsequent sintering at 1200 °C for 1 h were employed to obtain compact discs of ZNF*/*TCP composites.

Two series of ZNF/TCP composites were prepared. In the first series, Z2NF/TCP, Z4NF/TCP, Z6NF/TCP, and Z8NF/TCP composites containing 4 wt% of the respective ferrite were prepared for the preliminary cytocompatibility study. The second series contained varying amounts of Zn_0.8_Ni_0.2_Fe_2_O_4_ (Z8NF), ranging from 4 to 12 wt% in 2 wt% increments.

### Characterizations

Crystal structures were determined using an X-ray diffraction (XRD) system (PW-1830, Philips). Powder samples were analyzed using monochromatized CuK_*α*_ radiation over a 2*θ* range of 10°–60°, with a step size of 0.02° and a counting time of 8 s per step; the generator was operated at 30 kV and 25 mA. Rietveld refinement was carried out in TOPAS to determine the phase fractions. The background, lattice parameters, peak profile and scale factors were refined stepwise. The phase contents were obtained from the refined scale factors using the structural models.

Infrared spectral analysis was performed using a Fourier transform infrared (FTIR) instrument equipped with an attenuated total reflectance (ATR) accessory (PerkinElmer 2000). Data were collected at ambient conditions over the 400–2000 cm^-^¹ range.

The surface morphology of the sintered samples was investigated using a scanning electron microscope (SEM, Hitachi, S-4700) operated at a voltage of 10 kV. The open porosity was quantitatively evaluated by the liquid displacement method based on Archimedes’ principle.

Magnetic properties of composite specimens were determined using a vibrating sample magnetometer (VSM) at room temperature, with hysteresis loops recorded under an applied magnetic field of up to 9000 Oe.

Vickers hardness (HV) was measured using a microhardness tester (HMV-G21ST, Shimadzu). Indentations (*n* = 5) were made under a load of 5 N with a dwell time of 15 s.

### Cytocompatibility assays

The direct contact method was used to evaluate the response of human osteoblast (h-OB) cells on the ceramic surfaces following a routinely applied standard protocol at the National Metal and Materials Technology Center (MTEC, Thailand). All cell-based experiments were conducted at MTEC. Primary h-OB cells were isolated, characterized, and maintained at MTEC, and were provided for use in this study under the center’s ethical and biosafety guidelines. The cells were cultured in Dulbecco’s Modified Eagle’s Medium (DMEM) supplemented with 10% fetal bovine serum (FBS) and 1% penicillin–streptomycin (100 U/mL penicillin and 100 μg/mL streptomycin).

The test specimens, prepared as cylindrical pellets (1.5 cm in diameter and 0.5 cm in thickness) and sterilized by autoclaving at 121°C for 15 min, were soaked in culture medium and placed into 24-well plates for the biological assays. h-OB cells (1×10^4^ cells per sample) were seeded onto each specimen. The plates were incubated at 37 ± 1°C in a humidified atmosphere (95 ± 5% relative humidity) with 5 ± 0.1% CO₂ (pH 7.2–7.4) for 7 days.

Prior to SEM imaging, the cell-seeded specimens were fixed, dehydrated through a graded ethanol series, and sputter-coated with a thin layer of gold to improve surface conductivity. SEM observations were performed using a secondary electron (SE) detector, and representative micrographs were acquired at a magnification of 300×.

For the cytotoxicity MTT assay, the standard procedure outlined in ISO 10993-5 was followed. After the 7-day incubation period, 0.48 mL of MTT solution (0.5 mg/mL) was dispensed into each well, and the plates were incubated for a further 2 h. The specimens and culture medium were then discarded, followed by the addition of 0.8 mL of dimethyl sulfoxide (DMSO) to dissolve the formazan crystals. Subsequently, 100 µL of dye solution was transferred to each well, and the absorbance was measured using a microplate reader (Sunrise, Tecan, Switzerland) at 570 nm.

One-way analysis of variance (ANOVA) followed by the Ryan–Einot–Gabriel–Welsh (REGW) F multiple comparison test was used to compare the results. A *p*-value of less than 0.05 was considered statistically significant, corresponding to a 95% confidence level.

## Results and discussion

### *β*-TCP preparations

*β-*TCP was synthesized using calcium-deficient hydroxyapatite (calcium-deficient HAp; Ca_10‒*δ*_(HPO_4_)_*δ*_(PO_4_)_6‒*δ*_(OH)_2‒*δ*_, *δ* < 1) as a precursor. The calcium-deficient HAp was obtained via a precipitation reaction between Ca(NO_3_)_2_ and (NH_4_)_3_PO_4_, as described in Eq. (3). The Ca(NO_3_)_2_ precursor solution was derived from CaO sourced from chicken eggshells, as prepared according to Eq. (1) and (2).

1 $$\mathrm{CaC}{{\rm{O}}}_{3}\mathop{\to }\limits^{1100\,^\circ {\rm{C}}}\mathrm{CaO}+{\rm{C}}{{\rm{O}}}_{2}$$

2 $${\rm{CaO}}+{2{\rm{HNO}}}_{3}\to {\rm{Ca}}({\rm{N}}{{\rm{O}}}_{3}{)}_{2}+{{\rm{H}}}_{2}{\rm{O}}$$

3 $$9{\mathrm{Ca}}({\rm{N}}{{\rm{O}}}_{3}{)}_{2}+6({\rm{N}}{{\rm{H}}}_{4}{)}_{3}{\rm{P}}{{\rm{O}}}_{4}+{18\mathrm{NH}}_{4}{\mathrm{OH}}\mathop{\to }\limits^{100\,^\circ {\rm{C}}}{\mathrm{Ca}}_{9}({\mathrm{HPO}}_{4})({\mathrm{PO}}_{4}{)}_{5}(\mathrm{OH})+{18\mathrm{NH}}_{4}{\mathrm{NO}}_{3}$$

Biphasic calcium phosphate (BCP), consisting of *β-*tricalcium phosphate (*β-*TCP, *β*-Ca_3_(PO_4_)_2_; 310.18 amu per formula unit) and hydroxyapatite (HAp, Ca_10_(PO_4_)_6_(OH)_2_; 1,004.62 amu per formula unit), was obtained after calcination of the calcium-deficient HAp (Eq. (4)) [17].

4 $${\mathrm{Ca}}_{10-{\rm{\delta }}}{({\mathrm{HPO}}_{4})}_{{\rm{\delta }}}{({\mathrm{PO}}_{4})}_{6-{\rm{\delta }}}{(\mathrm{OH})}_{2-{\rm{\delta }}}\to 3{{\rm{\delta }}\mathrm{Ca}}_{3}{({\mathrm{PO}}_{4})}_{2}+(1-{\rm{\delta }}){\mathrm{Ca}}_{10}{({\mathrm{PO}}_{4})}_{6}{(\mathrm{OH})}_{2}+{{\rm{\delta }}{\rm{H}}}_{2}{\rm{O}}$$

Ideally, at the Ca/P of 1.5, Ca_9_(HPO_4_)(PO_4_)_5_(OH) was obtained (i.e. *δ* = 1) [18].

The calcination of Ca_9_(HPO_4_)(PO_4_)_5_(OH) for 3 h at 700 °C yielded only TCP:

5 $${\mathrm{Ca}}_{9}({\mathrm{HPO}}_{4}){({\mathrm{PO}}_{4})}_{5}(\mathrm{OH})\mathop{\to }\limits^{700\,^\circ {\rm{C}}}3{\mathrm{Ca}}_{3}({\mathrm{PO}}_{4})_{2}+{{\rm{H}}}_{2}{\rm{O}}$$

In practice, by controlling the Ca/P ratio at 1.5, a *δ* value approaching 1 was achieved, and the calcination product consisted predominantly of TCP. As an example, Eq. (6) shows the relative amounts of both phases of calcium phosphates after the calcination of HAp with *δ* = 0.99. TCP accounted for 98.92% and 99.46% of the total mass in calcined HAp with *δ* values of 0.99 and 0.995, respectively.

6 $${\mathrm{Ca}}_{9.01}{({\mathrm{HPO}}_{4})}_{0.99}{({\mathrm{PO}}_{4})}_{5.01}{(\mathrm{OH})}_{1.01}\to 2.97{\mathrm{Ca}}_{3}{({\mathrm{PO}}_{4})}_{2}+0.01{\mathrm{Ca}}_{10}{({\mathrm{PO}}_{4})}_{6}{(\mathrm{OH})}_{2}+0.99{{\rm{H}}}_{2}{\rm{O}}$$

**Fig. 2:**
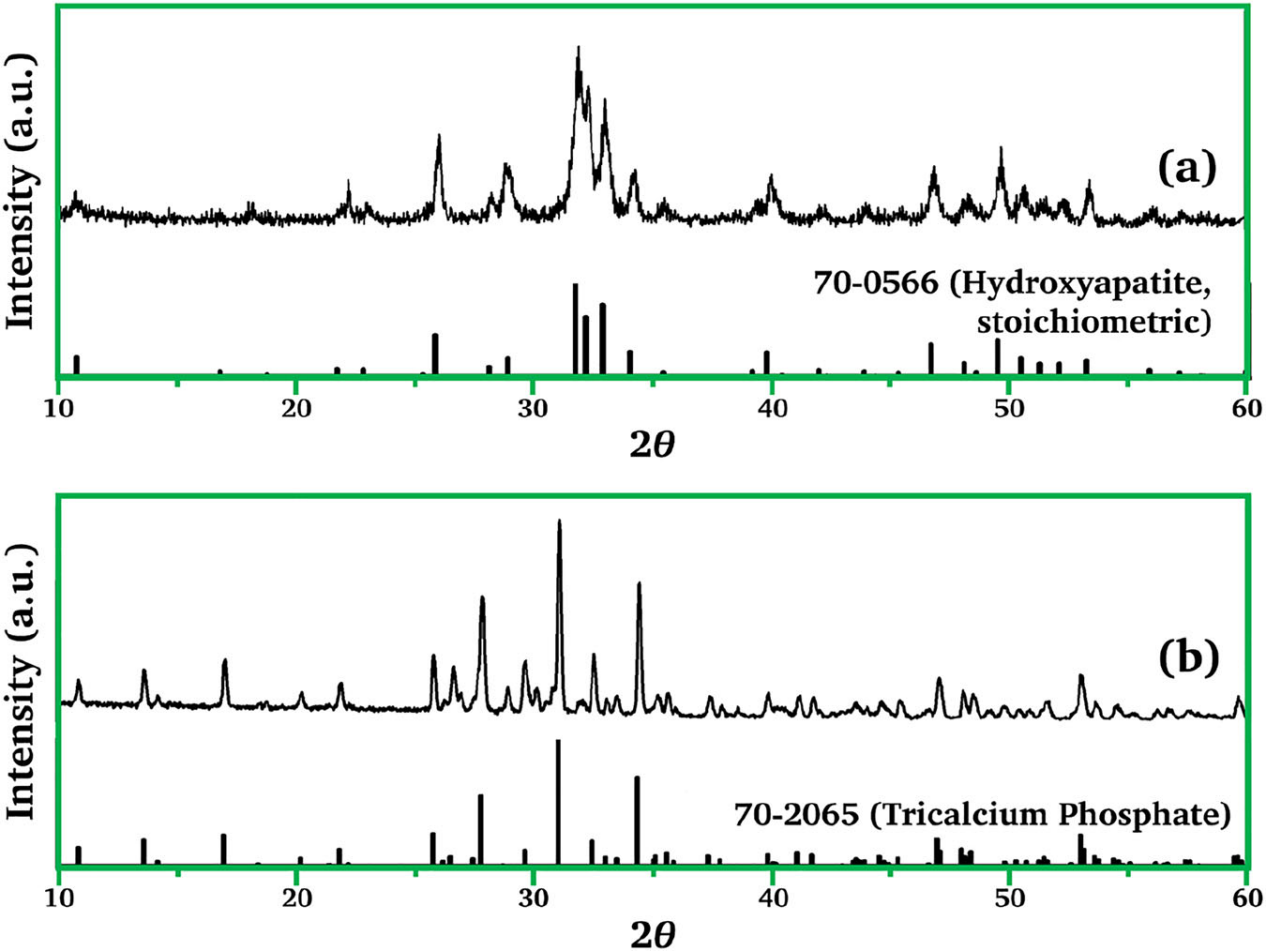
The XRD patterns of calcium-deficient hydroxyapatite (HAp): **a** prior to and **b** following calcination at 700 °C for 3 h

The XRD patterns of the as-synthesized product from Eq. (3) and the product after the calcination at 700 °C for 3 h are presented in Fig. 2. The main peaks of the as-synthesized product correspond to the (002), (210), (211), (112), (300), and (310) planes (Fig. 2a). It exhibited a poor crystal structure corresponding to a non-stoichiometric HAp phase (JCPDS file no. 70-0566). Figure 2b revealed that the calcined product was *β*-TCP (JCPDS file no. 70-2065). There are three crystalline polymorphs of TCP: *α*, *α’*, and *β*. The *α* and *α’* phases are stabilized under high temperature conditions. The XRD pattern also showed that a negligible amount of stochiometric HAp was present in the calcined product.

### ZNF preparations

Ferrites are typically composed of iron(III) oxide combined with one or more divalent metal oxides. The general stoichiometric formula for ternary ferrites (i.e., those containing iron oxide and one or more other metal oxides) is MFe_2_O_4_, where M denotes a divalent metal cation. In the present study, the Zn-Ni ferrites (ZNF) aimed for a target stoichiometric formula of (Zn,Ni)Fe_2_O_4_ (= Zn_*x*_Ni_1−*x*_Fe_2_O_4_), where *x* = 0.2, 0.4, 0.6, and 0.8. For simplicity, these ZNFs are referred to as ZxNF, e.g., Z2NF, Z4NF, Z6NF, and Z8NF. Co-precipitation was employed to prepare Zn–Ni–Fe hydroxide/oxide precursor mixtures with the desired molar ratios of Zn:Ni:Fe. The iron species were present predominantly as iron(III) oxyhydroxide (FeOOH), as represented in the reaction schemes below.

7 $$\begin{array}{l}(x)\mathrm{Zn}{({\mathrm{CH}}_{3}\mathrm{COO})}_{2}\cdot 2{{\rm{H}}}_{2}{\rm{O}}+(1-{\rm{x}})\mathrm{Ni}{({\mathrm{NO}}_{3})}_{2}\cdot 6{{\rm{H}}}_{2}{\rm{O}}+2\mathrm{Fe}{({\mathrm{NO}}_{3})}_{3}\cdot 9{{\rm{H}}}_{2}{\rm{O}}+8\mathrm{NaOH}\to \\ (x)\mathrm{ZnO}+(1-{\rm{x}})\mathrm{NiO}+2{\mathrm{FeOOH}}+(2x){\mathrm{CH}}_{3}\mathrm{COONa}+(8-2x){\mathrm{NaNO}}_{3}+(27-4x){{\rm{H}}}_{2}{\rm{O}}\end{array}$$

In an aqueous solution, Zn^2+^, Ni^2+^ and Fe^3+^ existed as 6-coordinated complexes, namely [Zn(CH_3_COO)_2_(H_2_O)_2_], [Ni(H_2_O)_6_]^2+^ and [Fe(H_2_O)_6_]^3+^, respectively. Therefore, the Eq. (7) may be better written as the reaction of the cationic coordinated metal complexes and hydroxide (OH^‒^) ions:

8 $$\begin{array}{l}(x)[\mathrm{Zn}{({\mathrm{CH}}_{3}\mathrm{COO})}_{2}{({{\rm{H}}}_{2}{\rm{O}})}_{2}]+(1-{\rm{x}}){[\mathrm{Ni}{({{\rm{H}}}_{2}{\rm{O}})}_{6}]}^{2+}+2{[\mathrm{Fe}{({{\rm{H}}}_{2}{\rm{O}})}_{6}]}^{3+}+8{\mathrm{OH}}^{-}\to \\ (x){\mathrm{ZnO}}+(1{-}{\rm{x}})\mathrm{NiO}+2{\mathrm{FeOOH}}+(2x){\mathrm{CH}}_{3}{\mathrm{COO}}^{-}+(21-4x){{\rm{H}}}_{2}{\rm{O}}\end{array}$$

For clarity, Eq. (7) is presented as a simplified and idealized representation of the co-precipitation process, in which the initially formed hydroxide and oxyhydroxide intermediates are expressed in their corresponding oxide/oxyhydroxide forms, as expected upon drying and subsequent calcination, since these species ultimately act as effective precursors for ferrite formation.

Calcination of the co-precipitated oxide/oxyhydroxide precursors at 800 °C yielded Zn-Ni ferrites (ZNFs) with the stoichiometric formula of (Zn,Ni)Fe_2_O_4_.

9 $$(x)\mathrm{ZnO}+(1-x)\mathrm{NiO}+2\mathrm{FeOOH}\mathop{\to }\limits^{800\,^\circ {\rm{C}}}{\mathrm{Zn}}_{x}{\mathrm{Ni}}_{1-x}{\mathrm{Fe}}_{2}{{\rm{O}}}_{4}+{{\rm{H}}}_{2}{\rm{O}}$$

Equation (9) represents an overall reaction scheme, in which the iron(III) oxyhydroxide (FeOOH) undergoes dehydroxylation to form Fe_2_O_3_ prior to and/or during calcination, followed by solid-state reaction with ZnO and NiO to yield the corresponding Zn–Ni spinel ferrite.

**Fig. 3:**
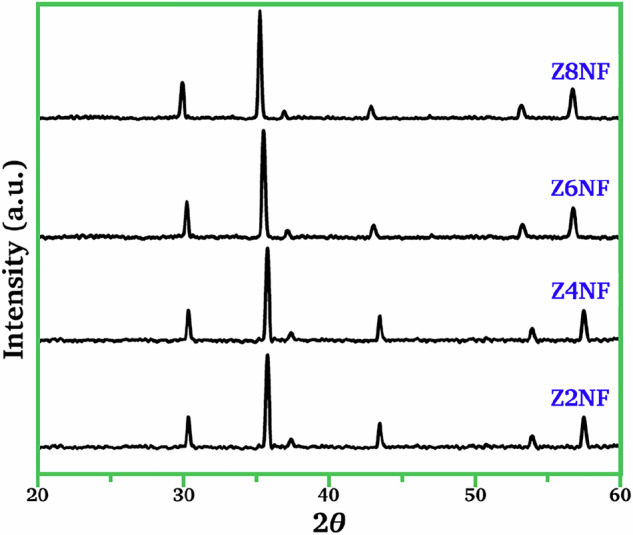
The XRD patterns for the crystalline Zn_*x*_Ni_1‒*x*_Fe_2_O_4_ obtained after calcination at 800 °C

The XRD patterns for the calcined co-precipitated ZnO/NiO/Fe_2_O_3_ are shown in Fig. 3. As expected, the solid-state reaction product exhibited major diffraction peaks indexed to the (220), (311), (400), (422), (511), (440) and (533) planes. The peaks correspond to a spinel structure and indicate the formation of ZNFs. The peak positions and intensities for all ZNFs were well-matched, and no impurities were indicated.

**Fig. 4:**
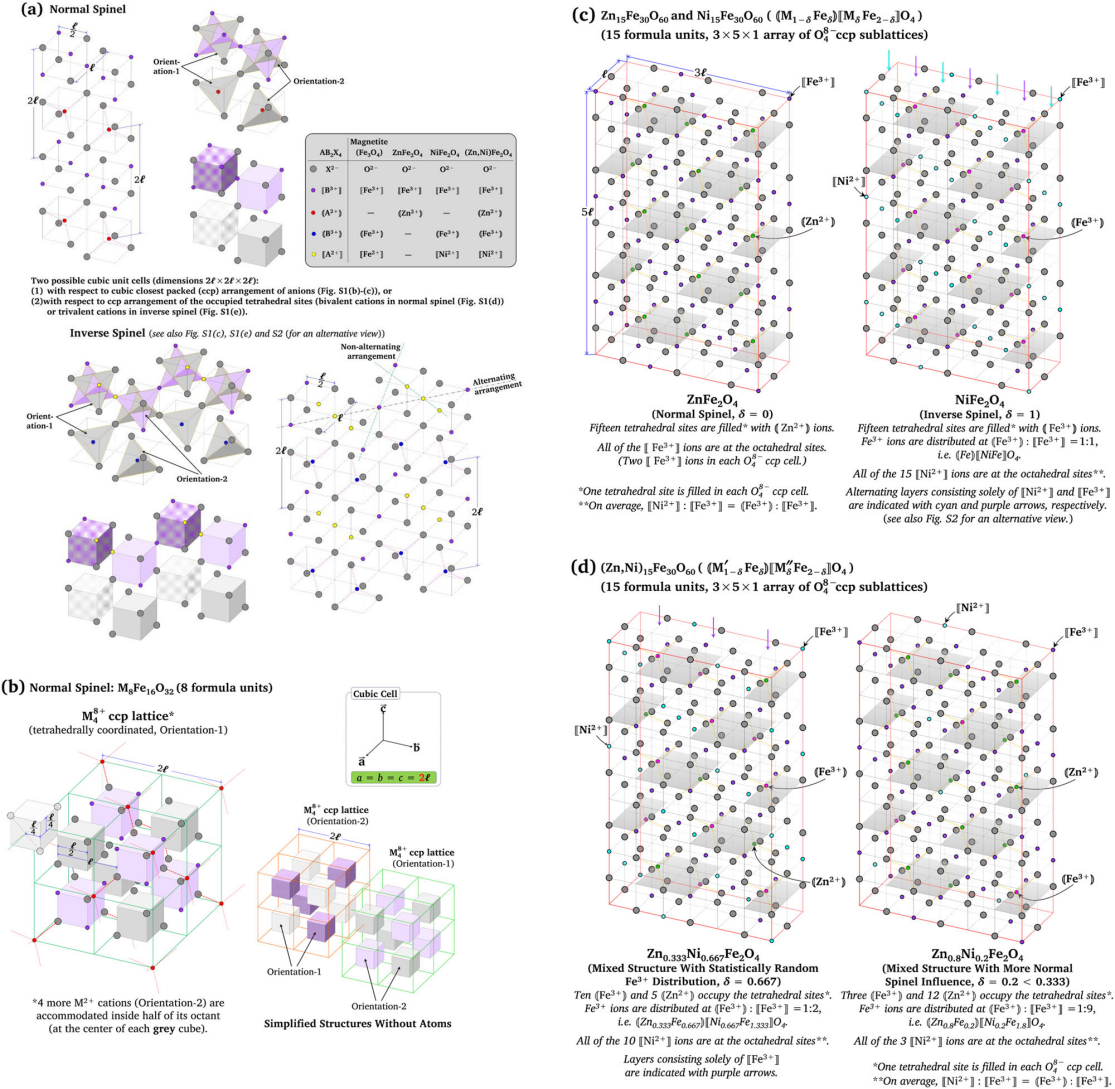
**a** minimal structure of cubic spinel, **b** cubic unit cells of normal spinel with respect to the ccp arrangement of divalent cations, **c** structure of Zn ferrite and Ni ferrite, and **d** structure of Zn-Ni ferrites. Atoms are not drawn to scale

Magnetite and ternary iron spinels (ferrites) crystallize in an AB_2_X_4_ cubic (isometric) crystal system where A, B, and X are typically a divalent cation, a trivalent cation, and a divalent anion, respectively (Fig. 4). In each AB_2_X_4_ formula unit (Figure S1a), there are 8 available tetrahedral sites (denoted with hollow parentheses, 〘 〙) and 4 available octahedral sites (denoted with hollow square brackets, 〚 〛). Within each (X^2‒^)_4_ cubic closest packed (ccp) anion sublattice, 1 of the 8 tetrahedral sites and 2 of the 4 octahedral sites are typically occupied. The minimal structures of spinel ferrites are shown in Fig. 4a, which illustrate two distinct arrangements of the oxygen–A^2+^ cubic sublattices and two distinct arrangements of the oxygen–B^3+^ cubic sublattices, together with their relative positions.

The cubic unit cell of ferrite contains 8 formula units (A_8_B_16_X_32_, 2×2×2 array of AB_2_X_4_) which can be viewed with respect to the cubic closest packed (ccp) arrangement of either the anions (oxygen, Fig. S1b for normal spinel) or the divalent cations (Fig. 4b for normal spinel, see also Fig. S1d). The structures of a typical inverse spinel are given in Fig. S1c and S1e. Figure 4a also shows the relationship between the 2 types of unit cells.

Magnetite and Ni ferrite typically crystallize as the inverse spinel structure, i.e. 〘Fe^3+^〙${\rm{[\kern-2pt[ }}$Fe^2+^Fe^3+^〛(O^2‒^)_4_ and 〘Fe^3+^〙${\rm{[\kern-2pt[ }}$Ni^2+^Fe^3+^〛(O^2‒^)_4_, respectively. The inverse spinel configuration arises from the strong octahedral-site preference of Ni^2+^ (in Ni ferrite) and the stabilization of Fe^2+^ at octahedral sites (in magnetite), thereby inducing a distribution of Fe^3+^ ions such that half of them occupy the tetrahedral sites (1 out of 8 sites in each formula unit) and the remainder occupy the octahedral sites (1 out of 4 sites in each formula unit; Fig. 4c, right). On the other hand, due to the strong preference of Zn^2+^ for tetrahedral sites, the Zn ferrite typically crystallizes as the normal spinel structure in which all of the Fe^3+^ ions are found exclusively in the octahedral sites (2 out of 4 sites in each formula unit), i.e. 〘Zn^2+^〙${\rm{[\kern-2pt[ }}$(Fe^3+^)_2_〛(O^2‒^)_4_ (Fig. 4c, left).

As a quaternary iron spinel with bivalent cations that exhibit preferences for either the tetrahedral or octahedral sites, the crystal structures of Zn_*x*_Ni_1‒*x*_Fe_2_O_4_ were expected to be an intermediate between the normal and inverse structures, i.e. 〘(Zn^2+^)_*x*_(Fe^3+^)_1−*x*_〙${\rm{[\kern-2pt[ }}$(Ni^2+^)_1−*x*_(Fe^3+^)_1+*x*_〛(O^2‒^)_4_. For a ternary iron spinel with Fe:O = 1:2, i.e. 〘(M^2+^)_1‒*δ*_(Fe^3+^)_*δ*_〙${\rm{[\kern-2pt[ }}$(M^2+^)_*δ*_(Fe^3+^)_2‒*δ*_〛(O^2‒^)_4_, an idealized statistically random cation distribution is obtained at the degree of inversion (*δ*) of ⅔ (0.667). There are 1 occupied tetrahedral site and 2 occupied octahedral sites but only 2 Fe^3+^ ions in each MFe_2_O_4_ formula unit. Therefore, the Fe^3+^ ions are distributed evenly over the 3 sites at *δ* = ⅔. In this way, the distribution of each cation equals the ratio of the occupied tetrahedral and octahedral sites, i.e. 〘M^2+^〙: 〚M^2+^〛 = 〘Fe^3+^〙 : 〚Fe^3+^〛 = 1:2. (Note that at *δ* ≠ 2/3, 〘M^2+^〙 : 〚M^2+^〛 ≠ 〘Fe^3+^〙 : 〚Fe^3+^〛).

In the case of a quaternary iron spinel ((M′,M″)Fe_2_O_4_, Fe:O = 1:2) with a complementary preference of site (with 〘M′^2+^〙 and 〚M″^2+^〛 predominantly occupying tetrahedral and octahedral sites, respectively), such as ZNF, the idealized statistically random Fe^3+^ distribution also occurs in ZNF with *δ* = 0.667 (*x* = 0.333; Z3.33NF), i.e. 〘(Zn^2+^)_0.333_(Fe^3+^)_0.667_〙${\rm{[\kern-2pt[ }}$(Ni^2+^)_0.667_(Fe^3+^)_1.333_〛(O^2‒^)_4_ (Fig. 4d, left). Therefore, the structure of Z8NF (〘(Zn^2+^)_0.8_(Fe^3+^)_0.2_〙${\rm{[\kern-2pt[ }}$(Ni^2+^)_0.2_(Fe^3+^)_1.8_〛(O^2‒^)_4_; Fig. 4d, right) is influenced more by the normal than the inverse structure, i.e. because of fewer 〘Fe^3+^〙 ions and higher 〚Fe^3+^〛 ions are present. The ratios of 〘 Fe^3+^〙 : 〚Fe^3+^〛 are ideally 1:1, 1:2, 1:9, and 0 in Ni ferrite/magnetite, Z3.33NF, Z8NF, and Zn ferrite, respectively.

### Cytocompatibility of 4-wt% ZNF/TCP

**Fig. 5:**
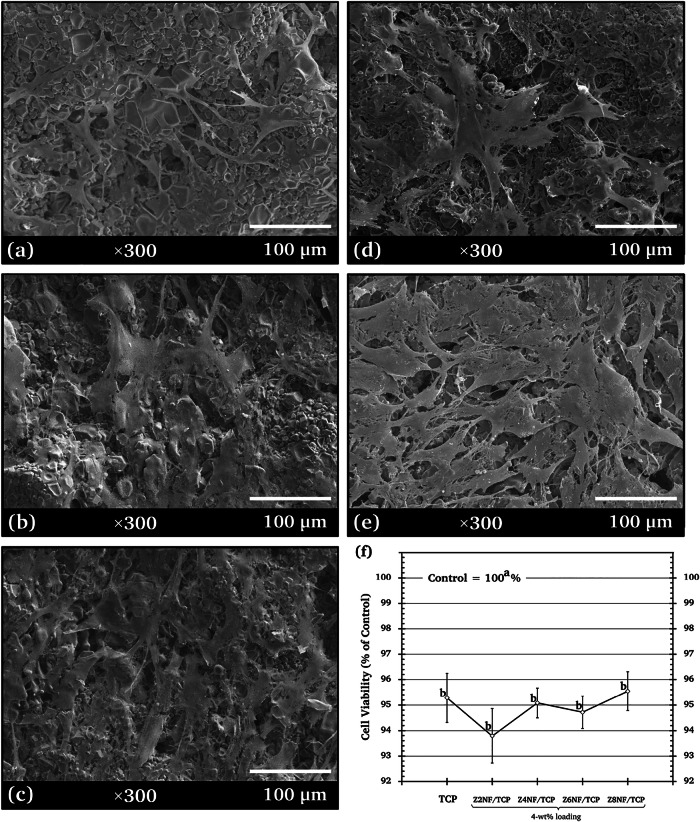
SEM micrographs (×300 magnification) showing cell morphology and density of h-OBs on surfaces of **a** *β*-TCP and the 4-wt% composites: **b** Z2NF/TCP, **c** Z4NF/TCP, **d** Z6NF/TCP, **e** Z8NF/TCP, and **f** MTT cell viability of h-OBs on *β*-TCP and 4-wt% ZNF/TCP composites after 7 days

The cytocompatibility of the ZNF/TCP composite ceramics was assessed based on the morphology and density of h-OB cells present on their surfaces, as well as quantitative cell viability. SEM images of h-OB cells cultured for 7 days on the specimens with 4-wt% ZNFs are shown in Fig. 5a–e, and Fig. 5f shows the corresponding 7-day cell viability assessed by MTT assay.

The extents of h-OB spreading on the surfaces of the tested composites were higher than those on the *β*-TCP ceramic (Fig. 5a). Cells on *β*-TCP showed a sparse distribution with limited spreading, whereas the ZNF/TCP composites supported clearer and denser cell coverage. The cells appeared broader, more elongated, and exhibited visible surface extensions, indicating better early attachment. Among the four compositions, Z8NF/TCP (Fig. 5e) showed the most extensive spreading, forming more continuous and connected cell layers across the surface. This behavior suggests that Z8NF/TCP provides a more favorable surface environment for initial h-OB attachment compared to the other samples. Thus, Z8NF/TCP (Zn_0.8_Ni_0.2_Fe_2_O_4_/*β*-TCP) was selected for further study of its physical properties, cell colonization, and cytotoxicity.

The MTT assay results indicated that all four 4-wt% composites (Z2NF/TCP, Z4NF/TCP, Z6NF/TCP, and Z8NF/TCP) exhibited similarly high cell viability with no significant differences, confirming that all formulations were non-cytotoxic according to ISO 10993-5 [19]. Because the quantitative viability data did not distinguish one composition from another [20], SEM observations were used solely as qualitative indicators of early cell attachment and surface interaction patterns to guide the selection of a representative composite for further investigation. Importantly, SEM was not used as a standalone measure of cytocompatibility but as a complementary tool to identify the sample with the most favorable early attachment behavior. Based on its broader spreading, denser coverage, and more continuous surface layers, Z8NF/TCP was therefore selected for subsequent characterization.

To place these SEM observations in context, the limitations of SEM and the complementary quantitative results were also considered. While SEM images provide qualitative evidence of cell attachment and distribution, they cannot be used to quantitatively assess adhesion or cytocompatibility [21].

These findings are consistent with previous studies that have emphasized the qualitative nature of SEM-based evaluation. Brochado et al. [22] demonstrated that SEM can visualize early cell spreading and surface interaction patterns in osteosphere-based models, while Voelkner et al. [23] reported that SEM reveals osteoblast surface morphology and membrane protrusions but cannot quantify adhesion strength. Korkeamäki et al. [24] used SEM to assess cell distribution and attachment on porous scaffolds, and Gutiérrez Prieto et al. [25] showed that SEM can depict osteoblast morphology, including surface extensions associated with attachment. Therefore, although SEM provides preliminary qualitative insight into h-OB spreading on the composite surfaces, more quantitative and reliable methods—such as fluorescent staining of nuclei and cytoskeleton—should be incorporated in future studies to validate adhesion and morphological responses.

### Phases in Z8NF/TCP composites

**Fig. 6:**
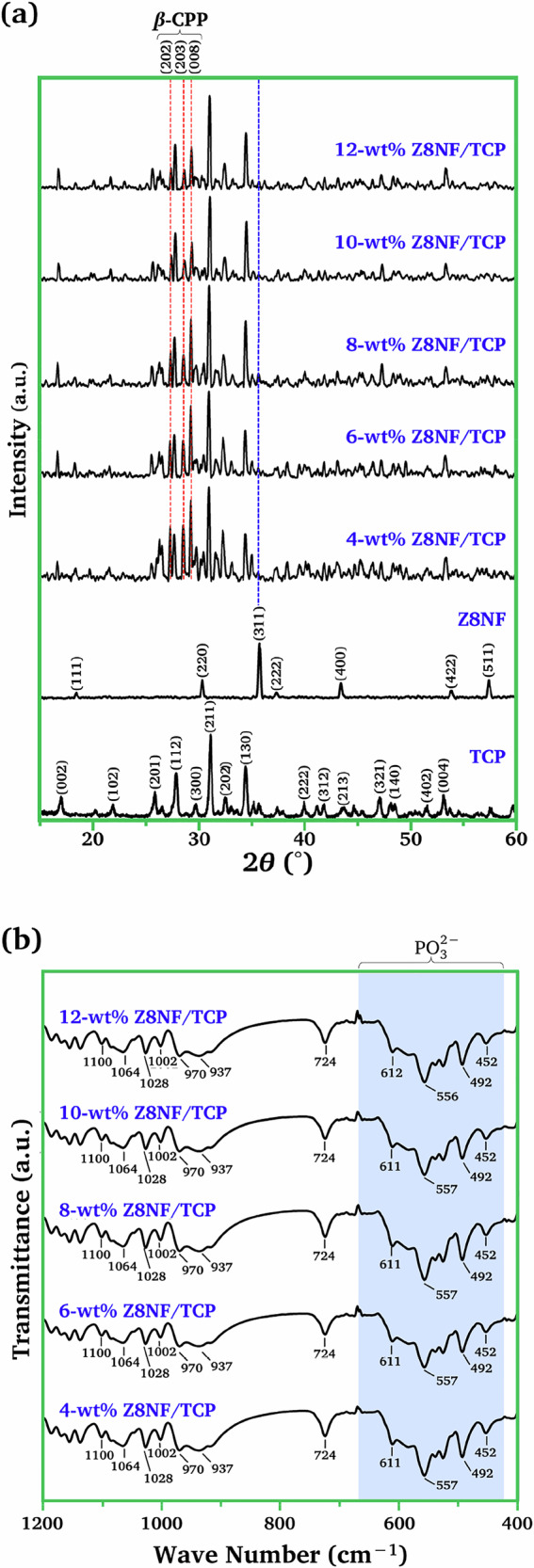
**a** The XRD patterns and **b** ATR-FTIR of 4–12 wt% Z8NF/TCP

Figure 6a presents the XRD analysis results for the 4–12 wt% Z8NF/TCP compared to those of the two components in isolation. The *β*-TCP and Z8NF phases can be identified along with *β*-calcium pyrophosphate (*β*-Ca_2_P_2_O_7_; *β*-CPP) consistent with the *β*-TCP → *β*-CPP transformation during sintering. All Z8NF/TCP composites were subjected to full peak-matching analysis. This pattern confirms the presence of only three crystalline phases—*β*-TCP, Z8NF, and *β*-CPP—which were consistently identified across all compositions. In addition, all Z8NF/TCP composites underwent Rietveld refinement to determine the phase fractions.

A small peak corresponding to the (311) plane of the Z8NF phase was observed; however, its intensity was weak, likely due to partial overlap with *β*-TCP reflections and the relatively low Z8NF content in the composite, particularly in the 25–35° region where weak Z8NF reflections coincide with the dense Ca–P diffraction peaks.

The new *β*-CPP phase was assigned to the major peaks of the (202), (203) and (008) planes, as indexed in JCPDS file no. 09-0346. In addition, several weak minor reflections consistent with *β*-CPP were also identified based on the full peak-matching analysis, further supporting the formation of the *β*-CPP phase in the composites.

To confirm the occurrence of *β*-CPP phase in the composites, the ATR-FTIR study was carried out and the spectra are reported in Fig. 6b. The spectra for the Z8NF/TCP composites of all weight ratios exhibited similar patterns. Pyrophosphate or diphosphate (P_2_O_7_^4‒^) is the product of the condensation of two phosphate (PO_4_^3−^) groups, consisting of two PO_4_^3−^ groups linked by a central oxygen atom. The major bands relating to the PO_4_^3−^groups of *β*-CPP were observed around 452, 492, 557, and 611 cm^‒1^, and agreed well with previously reported spectra of pure *β*-CPP [26, 27]. The FTIR profile reported by Griesiute et al. [27] also shows characteristic PO₄³⁻ bending vibrations within the 450–620 cm⁻¹ region, supporting the assignments made in this study. Minor shifts in these bands may arise from variations in crystallinity, lattice strain, partial Zn²⁺/Ni²⁺ substitution, or residual hydration, which are known to influence phosphate vibrational modes in calcium phosphate systems [28]. Although pure *β*-CPP was not measured directly in this work, the combined evidence from XRD phase matching and literature-validated FTIR fingerprints provides a consistent basis for confirming *β*-CPP formation in the composites.

The other vibrational bands corresponded to those of the *β*-TCP phase. No distinct Z8NF bands were observed in the spectra, likely because the low-intensity Fe–O/Zn–O/Ni–O vibrations of the Z8NF spinel were obscured by the stronger phosphate vibrational bands of *β*-TCP and *β*-CPP, a phenomenon commonly noted in calcium phosphate systems [28].

Table 1 summarizes the quantitative phase fractions of the Z8NF/TCP composites. As the Z8NF content increased, the *β*-TCP fraction progressively decreased, whereas the *β*-CPP fraction increased accordingly, consistent with the *β*-TCP → *β*-CPP transformation observed in the XRD patterns. The Z8NF phase remained a minor component (0.46–1.73 wt%) across all compositions due to its low initial loading and the partial overlap of its weak reflections with the dominant *β*-TCP peaks.

**Table 1:** Quantitative composition of *β*-TCP, *β*-CPP, and Z8NF (Zn_0.8_Ni_0.2_Fe_2_O_4_) phases in Z8NF/TCP composites, along with the porosity of each composite

Samples	Phase Composition (wt%)			Porosity (%)
	*β*-TCP	*β*-CPP	Z8NF	
4-wt% Z8NF/TCP	68.86	30.68	0.46	33.3
6-wt% Z8NF/TCP	57.12	41.15	1.73	38.5
8-wt% Z8NF/TCP	50.26	48.16	1.58	42.3
10-wt% Z8NF/TCP	39.06	60.06	0.88	44.7
12-wt% Z8NF/TCP	34.97	63.73	1.30	45.6

The *β*-CPP reflections were first confirmed through full-profile peak matching (JCPDS 09-0346), ensuring that the assignment used in the Rietveld refinement is accurate. It is important to note that the phase fractions obtained from the Rietveld refinement are determined from the integrated peak intensities of the entire diffraction pattern rather than from the apparent peak heights observed in the qualitative XRD profiles. Within the 20–35° region, substantial overlap among the *β*-CPP, *β*-TCP, and Z8NF reflections—combined with peak broadening and differences in crystallinity—can give rise to a visual decrease in the *β*-CPP peak height at higher Z8NF contents. Nevertheless, the whole-pattern fitting approach used in the refinement accurately deconvolutes these contributions, thereby explaining why the refined *β*-CPP fraction increases even though the corresponding peaks appear reduced in height.

It should be clarified that the Z8NF values reported in Table 1 correspond to the crystalline, XRD-detectable fraction quantified by Rietveld refinement (i.e., normalized to the refined crystalline phases), and therefore do not necessarily equal the nominal ferrite addition (4–12 wt%). In multiphase Ca–P matrices, ferrite can be highly dispersed as nanoscale domains and/or occur as poorly crystalline interfacial components, which contribute weak and broadened features and may therefore be underestimated by XRD-based phase quantification. This limitation is further amplified by the severe overlap between the weak Z8NF reflections (notably the (311) peak) and the dense *β*-TCP/*β*-CPP peak population in the 25–35° region. Importantly, peak matching and Rietveld analysis consistently identified only *β*-TCP, *β*-CPP, and Z8NF across all compositions, with no evidence of additional crystalline ferrite-derived phosphates/oxides or Ca-rich crystalline phases, indicating that Z8NF primarily persists as a stable secondary phase while promoting the *β*-TCP → *β*-CPP transformation through an interfacial-stress-assisted solid-state mechanism rather than being extensively consumed during sintering.

The *β*-TCP → *β*-CPP transformation is further confirmed quantitatively by the reduction in *β*-TCP from 68.86% to 34.97% and the corresponding increase in *β*-CPP to 63.73%. This interpretation is supported by its nearly unchanged phase fraction, the absence of Z8NF-derived phosphate phases in the XRD patterns, and the stable ATR-FTIR signatures.

The observed phase evolution is governed by localized lattice distortion and thermally induced recrystallization within the *β*-TCP structure during high-temperature sintering [29]. The incorporation of Z8NF introduces interfacial stress that perturbs the *β*-TCP lattice, promotes redistribution of Ca–P structural units, and enhances the *β*-TCP → *β*-CPP transformation.

The transformation can be described by the solid-state reaction:

10 $$\beta \hbox{-} {{\rm{Ca}}}_{3}{({{\rm{PO}}}_{4})}_{2}\to \beta \hbox{-} {{\rm{Ca}}}_{2}{{\rm{P}}}_{2}{{\rm{O}}}_{7}+{\rm{CaO}}$$

In practice, any CaO generated during the *β*-TCP → *β*-CPP transformation is unlikely to persist as free CaO after sintering. Previous studies have shown that phosphate evaporation during *β*-TCP sintering can lead to the formation of Ca-rich surface or grain-boundary species, which readily transform into hydroxide, carbonate, or amorphous Ca-rich boundary phases upon cooling and exposure to air. In addition, segregation of Ca and its interaction with the crucible or surrounding environment may result in trace calcium-bearing silicate or aluminate glassy phases. Such phases, if present, would be amorphous and below the detection limit of conventional XRD, yet they could still influence local mass transport and diffusion processes, thereby affecting the observed phase evolution during sintering [30].

Accordingly, the increase in *β*-CPP content correlates well with the rise in porosity from 33% to 46%, suggesting that both *β*-CPP formation and the presence of finely dispersed Z8NF crystallites hinder densification and promote pore retention during sintering. In addition, the presence of Ca-rich grain-boundary or amorphous interfacial phases, potentially formed via phosphate evaporation and Ca segregation at high temperatures, may additionally impede mass transport and grain-boundary diffusion, thereby contributing to the observed inhibition of densification and increased pore retention.

In our system, there is no evidence for the formation of additional calcium ferrite, magnetite, or other ferrite-derived Ca-rich crystalline phases; only *β*-TCP, *β*-CPP, and Z8NF were consistently detected by XRD and confirmed by Rietveld phase analysis.

Rather than extensive ionic substitution, the available data indicate that any incorporation of Zn^2+^, Ni^2+^, or Fe^3+^ into the *β*-TCP lattice, if present, is negligible. This is consistent with the crystallochemical constraints on Ca^2+^ sites in *β*-TCP reported by Bigi et al. [31], as well as with the low Z8NF loadings used in this work. Instead, Z8NF persists as a stable spinel ferrite secondary phase that generates interfacial strain and thermal-expansion mismatch during sintering. These interfacial stresses destabilize the *β*-TCP framework and facilitate the redistribution of Ca–P units, thereby accelerating the *β*-TCP → *β*-CPP transformation through a solid-state mechanism, in agreement with the sintering behavior described by Champion [32].

*β*-CPP is well regarded for its osteoinductive potential and its ability to support early cellular responses, offering faster and more controlled degradation than HAp or *β*-TCP [33]. Its resorption releases bioactive pyrophosphate and orthophosphate species that promote osteoblast differentiation and matrix mineralization, enabling earlier tissue maturation [33]. Recent preclinical and clinical findings also show that *β*-CPP ceramics undergo continuous resorption accompanied by new bone formation, reinforcing their effectiveness as biodegradable bone graft substitutes [33, 34].

Although *β*-CPP has been widely reported to promote osteogenic activity and accelerate bone regeneration, its biological response is highly context-dependent. In particular, inflammatory reactions have mainly been associated with uncontrolled CPP impurities or particulate debris that induce excessive local ion release and macrophage activation, rather than with structurally integrated and compositionally controlled *β*-CPP phases in bulk scaffolds, as demonstrated in previous studies focusing on CPP-related inflammatory responses [35, 36].

Together, these findings highlight that Z8NF/TCP composites benefit from the complementary functions of *β*-TCP and *β*-CPP. The *β*-TCP phase provides structural support and moderate resorption, while the *β*-CPP fraction contributes faster ion release and enhanced osteogenic activity. This dual-phase synergy enables controllable degradation and tailored biological responses suited for targeted biomedical applications. Nevertheless, further in vivo investigations focusing on inflammatory and immune responses will be essential to fully validate the clinical safety of the proposed scaffold design.

### Surface morphologies, porosities, and hardnesses of composites

**Fig. 7:**
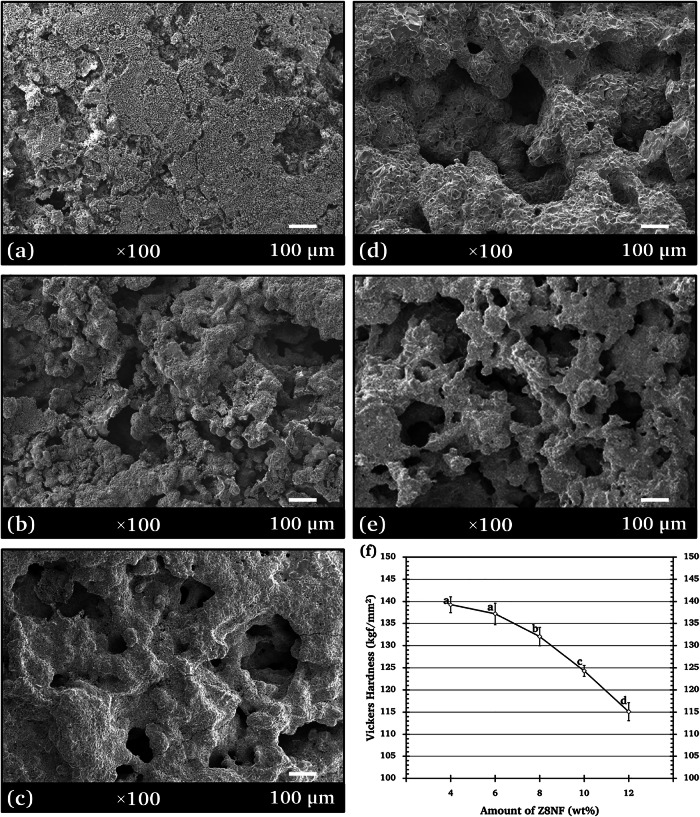
SEM micrographs (x100 magnification) illustrating the surface morphology of Z8NF/TCP composites consisting of **a** 4-wt%, **b** 6-wt%, **c** 8-wt%, **d** 10-wt%, and **e** 12-wt% Z8NF. **f** Vickers hardness values measured for each Z8NF amount

SEM images of the Z8NF/TCP (Fig. 7a‒e) revealed that the composites were microporous structures exhibiting polygonal grain morphologies. Interconnections between these pores throughout the composite facilitate bone ingrowth, osteoconduction, and the exchange of nutrients and oxygen, thereby promoting vascularization. These properties contribute to effective osseointegration and long-term stability, supporting its potential as a biomaterial [37]. Moreover, macropores also support cell seeding, distribution, migration and further neovascularization in vivo [38, 39].

The porosity of the composite increased from 33.3% to 45.6% with increasing Z8NF amount from 4 wt% to 12 wt% (Table 1). Because the porosity level and interconnectivity of the composites increased with higher Z8NF weight ratios, it is almost certain that the 12-wt% Z8NF/TCP exhibited the highest surface area. A structure exhibiting high porosity and surface area facilitates attachment, growth, proliferation and differentiation of cells [40]. The significantly increased porosity is consistent with the reduced sinterability of composites containing higher Z8NF content, as discussed in Section “Phases in Z8NF/TCP composites”.

The Vickers hardnesses of the composites are given in Fig. 7f. The hardnesses decreased as the Z8NF content increased. The increases in the porosities with the Z8NF content may have induced more bulk defects, thereby reducing the applied stress resistance and decreasing the hardnesses. Similar results have been reported in the literature, indicating that the porosity significantly influences the hardness of ceramics [41, 42].

While this might suggest lower structural strength, the values remained within an acceptable range for temporary bone scaffolds. Importantly, the reduced hardness did not compromise the composite’s handling or its ability to support cell proliferation [43].

### Magnetic behaviors of composites

**Fig. 8:**
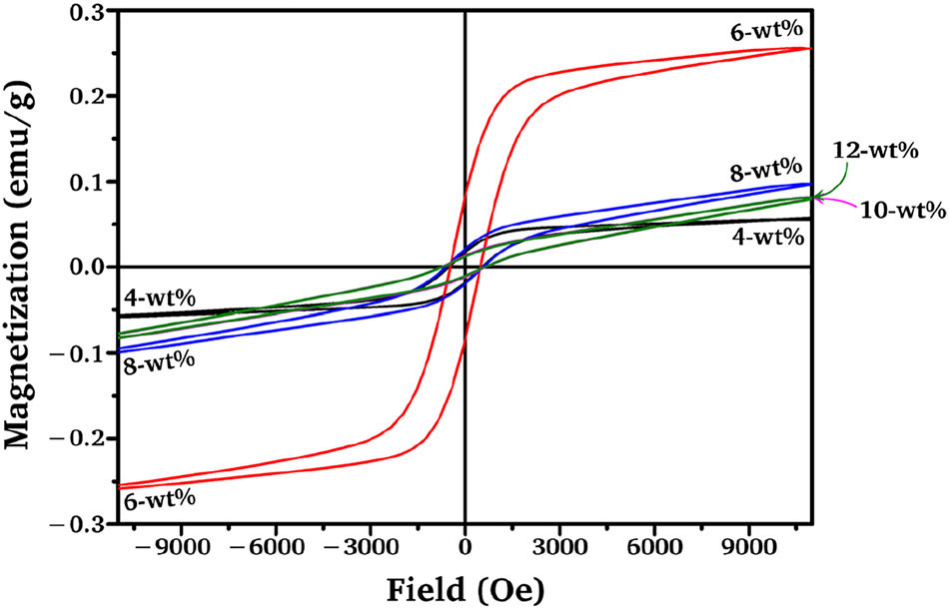
VSM hysteresis loops for the Z8NF/TCP composites

Introducing Z8NF provided soft magnetic characteristics to the composite. The VSM hysteresis loops of the Z8NF/TCP composites are given in Fig. 8. Noted a close resemblance between the hysteresis loops of 10-wt% (pink) and 12-wt% of Z8NF/TCP (green) composites and, as a result, the loop of the former was almost completely masked by that of the latter in Fig. 8.

The saturation magnetizations (*M*_s_) were 0.058, 0.259, 0.101, 0.085, and 0.084 emu/g for 4‒12 wt% Z8NF/TCP, respectively. Although the 4-wt% composite has the lowest porosity (33.3%), it shows the lowest *M*_s_ because its Z8NF phase fraction is only 0.46 wt%, insufficient to form continuous magnetic pathways. In this composition, the ferrite phase remains as isolated clusters within the *β*-TCP/*β*-CPP matrix, resulting in weak domain coupling and limited magnetization.

When the Z8NF content increases to 6 wt%, the ferrite phase fraction (1.73 wt%) becomes sufficient to form an interconnected magnetic network, yielding the highest *M*_s_ despite its higher porosity. At higher loadings (8–12 wt%), the combined effects of increased porosity and dilution by the non-magnetic *β*-TCP/*β*-CPP matrix progressively disrupt magnetic connectivity, reducing *M*_s_ accordingly.

These results confirm that the magnetic behavior of Z8NF/TCP composites is governed primarily by ferrite percolation and pathway connectivity, rather than porosity alone.

The corresponding coercivities (*H*_c_) were 528.25, 479.31, 587.77, 694.14, and 678.12 Oe, respectively. The higher porosities in the structure may have increased the effective magnetic anisotropy, resulting in enhanced *H*_c_ values.

Earlier studies have shown that moderate magnetism, even with low magnetization, can positively affect bone cell activity through mechanisms such as enhanced protein adsorption or ion mobility under magnetic influence [44−46].

Tran and Webster [47]. and Zeng et al. [48] conducted interesting studies on the biological activities of magnetic HAp composites with low saturation magnetizations. They found that synthesized magnetic HAp nanoparticles enhanced collagen synthesis, calcium mineralization, and alkaline phosphatase levels, indicating improved osteoblastic function supporting bone regeneration. Zeng et al. [48] developed a magnetic HAp scaffold that stimulated cellular attachment, growth, and cell differentiation better than a pure HAp scaffold because of the intrinsic magnetic behavior of the combined magnetic material. Moreover, these scaffolds responded well to an external magnetic field.

Fe_3_O_4_ (magnetite) is considered a weak hard ferrite, exhibiting moderate coercivity and remanent magnetization, while ZNF is a typical soft ferrite, characterized by low coercivity, low hysteresis loss, and moderate magnetic response.

Soft ferrites, including Zn-Ni-substituted ferrites, are advantageous in biomedical fields for several reasons. Soft ferrites exhibit negligible energy loss during magnetization and demagnetization cycles. This is crucial for applications in which repeated exposure to an alternating magnetic field is involved. Such applications include targeted drug delivery, bone regeneration, and magnetic hyperthermia. Unlike some hard ferrites or metallic magnetic nanoparticles, soft ferrites tend to exhibit better biocompatibility and reduced oxidative stress, minimizing damage to surrounding tissues or cells. Moreover, the moderate *M*_s_ of soft ferrites allows precise control under external magnetic fields without risk of strong agglomeration or heating. This property is essential in scaffold-based bone regeneration or MRI contrast agents [49].

In contrast, Fe_3_O_4_ is less favorable in applications requiring fine control of magnetic response or minimal thermal effects due to the inherent properties of hard ferrites even though it is biocompatible and widely studied. Thus, the use of ZNF soft ferrite to prepared magnetic TCP-based bioceramic not only offers structural and biological advantages but also ensures magnetic safety and functional precision that aligns with the demands of modern bone tissue engineering and magnetically-responsive biomaterials.

### Cell colonization and MTT assay

**Fig. 9:**
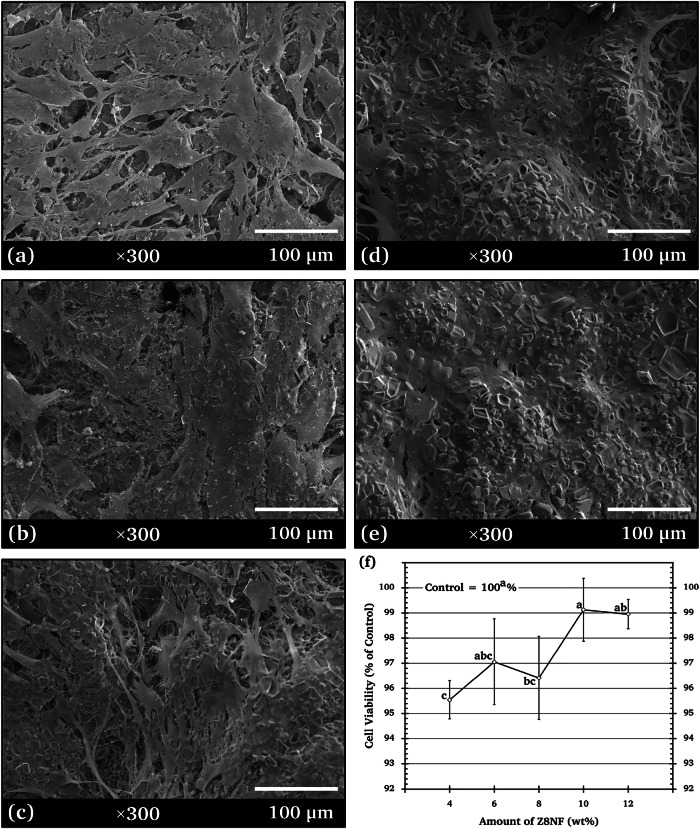
SEM micrographs (×300 magnification) showing the morphologies and densities of h-OB cells on the Z8NF/TCP composite surfaces consisting of **a** 4-wt%, **b** 6-wt%, **c** 8-wt%, **d** 10-wt%, and **e** 12-wt% Z8NF, and **f** cell viability (cytotoxicity MTT assay) after 7 days of incubation measured for each Z8NF amount

Examinations of the h-OB cell morphology and density on Z8NF/TCP composite surfaces following 7 days of incubation (Fig. 9a‒e) showed that the h-OB cells attached firmly and spread well over the surfaces and infiltrated the porous structure of all the tested specimens. These observations indicate that all Z8NF/TCP composites provide a favorable surface for h-OB attachment and colonization. More extensive colonization of h-OB cells was observed on the surfaces of 8‒12 wt% Z8NF/TCP. The SEM images have shown that ceramic structures with high surface area and roughness directly affect cell activities by providing sites for cell adhesion and growth [50]. Xue et al. [51] reported a similar result that a structure with large (200 μm) pores enabled cell ingrowth. The composite ceramic developed in this study, which exhibited increased macroporosity and surface complexity with higher Z8NF contents, is expected to have a high protein adsorption capacity, which promotes cell accommodation and proliferation.

Figure 9f shows the 7-day h-OB cell viabilities measured by MTT assay. No statistically significant differences were observed among the Z8NF/TCP composites and the *β*-TCP control, indicating that all compositions were non-cytotoxic according to ISO 10993-5 and demonstrating good cytocompatibility.

The improved h-OB colonization observed in the Z8NF/TCP composites, particularly in those with 8–12 wt% Z8NF, resulted from the combined influence of several interrelated material characteristics. The increase in porosity and pore interconnectivity with higher Z8NF contents likely enhanced the surface area available for cell attachment and nutrient diffusion which are factors reported to support osteoconduction and bone tissue formation [52, 53]. In addition, the progressive increase in *β*-CPP phase fraction (30.68 → 63.73 wt%) with higher Z8NF contents may further contribute to early osteogenic responses, as *β*-CPP releases phosphate species known to support osteoblast activity [50, 51].

## Conclusion

Composites of zinc–nickel ferrite and *β*-tricalcium phosphate (ZNF/TCP) were successfully fabricated via a solid-state reaction route using *β*-TCP derived from chicken eggshells and co-precipitated Zn–Ni ferrite powders. All composites showed good cytocompatibility, with enhanced human osteoblast (h-OB) attachment relative to pure *β*-TCP. Among the four ferrite formulations investigated at 4 wt%, the Zn_0.8_Ni_0.2_Fe_2_O_4_-containing composite (Z8NF/TCP) promoted the most extensive cell spreading and was therefore selected for further study.

For Z8NF/TCP composites containing 4–12 wt% Z8NF, all samples supported h-OB attachment, surface spreading, and pore infiltration. The greatest extents of surface colonization occurred in the 8–12 wt% composites. MTT assays confirmed non-cytotoxicity across all compositions, with cell viabilities comparable to *β*-TCP.

High-temperature sintering induced partial *β*-TCP → *β*-CPP (*β*-Ca_2_P_2_O_7_) transformation, as confirmed by XRD and ATR-FTIR. Increasing Z8NF content progressively enhanced *β*-CPP formation and elevated composite porosity while reducing densification and Vickers hardness. Z8NF remained a minor but stable secondary phase ( < 2 wt%) and did not participate in Ca–P lattice substitution.

Magnetic characterization revealed that the *M*_s_ was controlled primarily by ferrite percolation and magnetic-pathway connectivity rather than by bulk Z8NF loading. The 6-wt% Z8NF, which contained the highest ferrite phase fraction (1.73 wt%), exhibited the greatest *M*_s_, while higher Z8NF loadings (8–12 wt%) showed reduced *M*_s_ due to increased porosity and dilution by the non-magnetic *β*-TCP/*β*-CPP matrix. Higher *H*_c_ values were observed for the more porous composites, particularly at higher Z8NF loadings (8–12 wt%), reflecting enhanced effective magnetic anisotropy associated with the porous microstructure.

Overall, the Z8NF/TCP composites integrate (i) bioactivity from *β*-TCP, (ii) a potential osteogenic-supporting contribution from *β*-CPP, and (iii) controllable soft-magnetic response from Z8NF. The combined structural, magnetic, and biological properties highlight their potential as multifunctional biodegradable bioceramics for bone-reinforcing and bone-regeneration applications.

## Supplementary information

Supplementary information
