# Supplementary material for: Fabrication and characterization of biodegradable Zn-Ni spinel ferrite/ β-TCP composite ceramics exhibiting enhanced cell colonization
Source: J Mater Sci Mater Med. 2026 Jan 29;37(1):31. doi: 10.1007/s10856-026-07004-7 (PMC12864323; doi:10.1007/s10856-026-07004-7)
Supplement: Supplementary file 1 — Supplementary information [file 10856_2026_7004_MOESM1_ESM.pdf]

Supplement

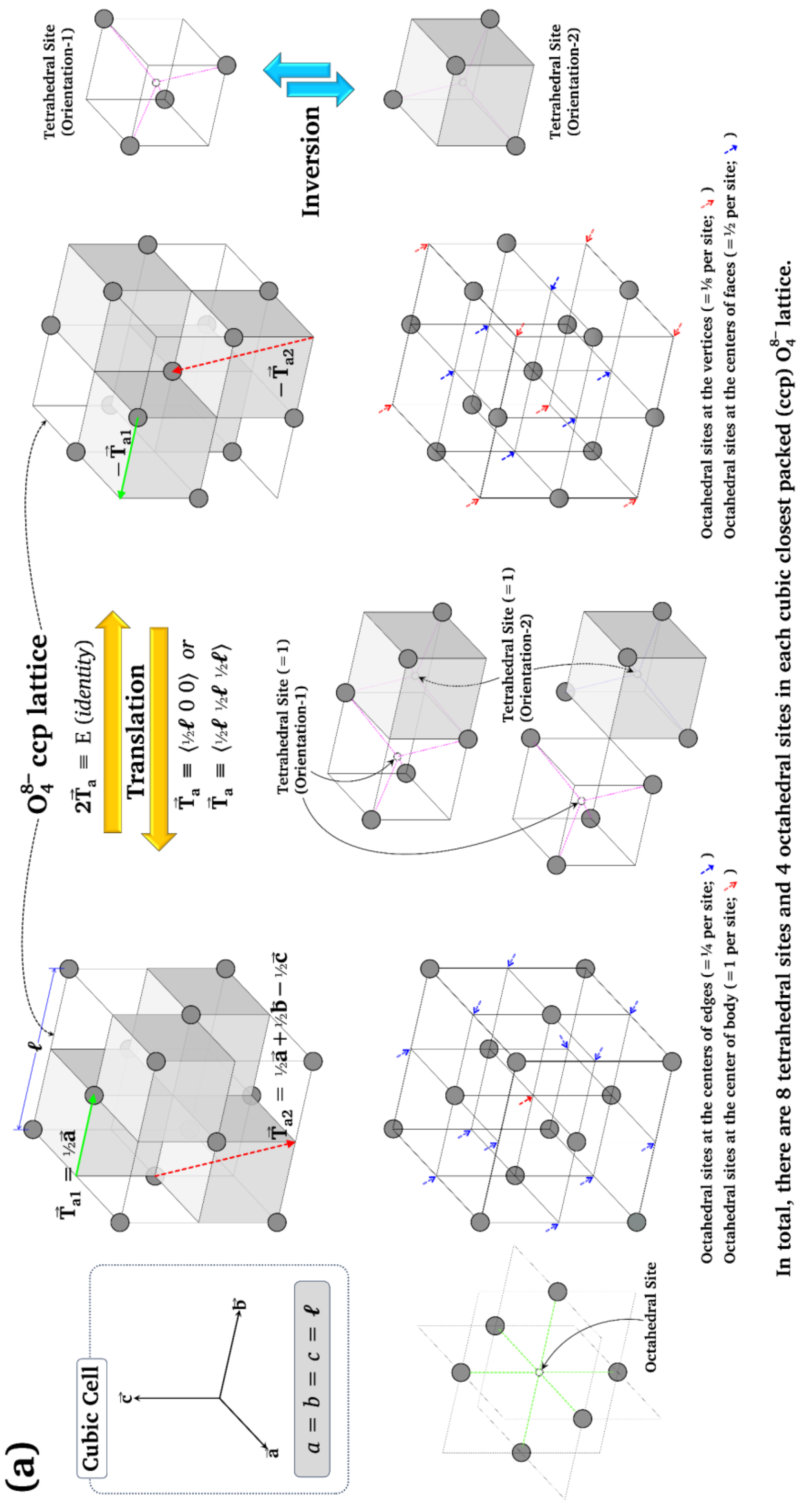

Fig. S1 (a) cubic closest packed (ccp) lattice of oxygen atoms showing the tetrahedral and octahedral sites.

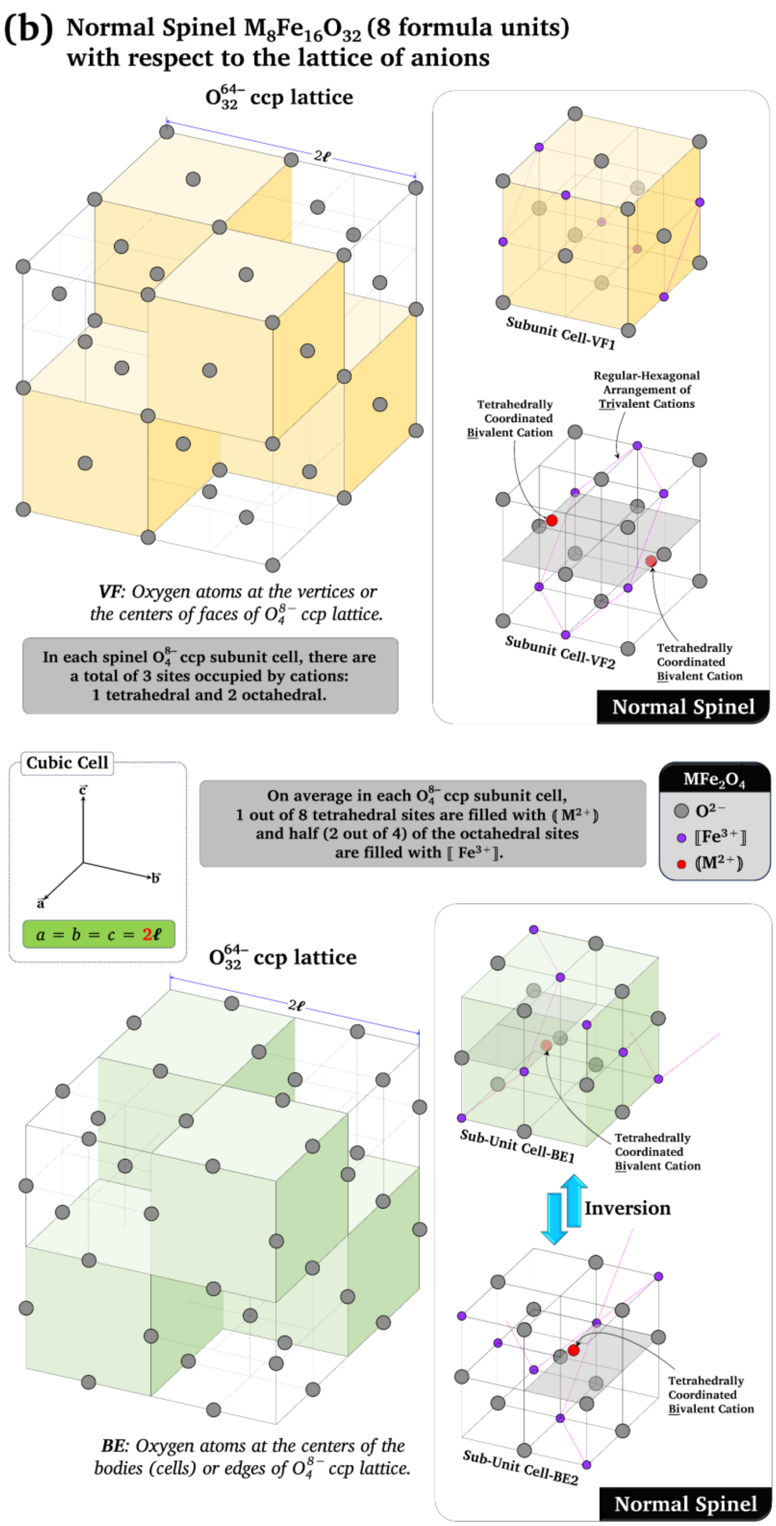

Fig. S1 *continued* (b) cubic unit cells of normal spinel with respect to the ccp arrangement of the anions.

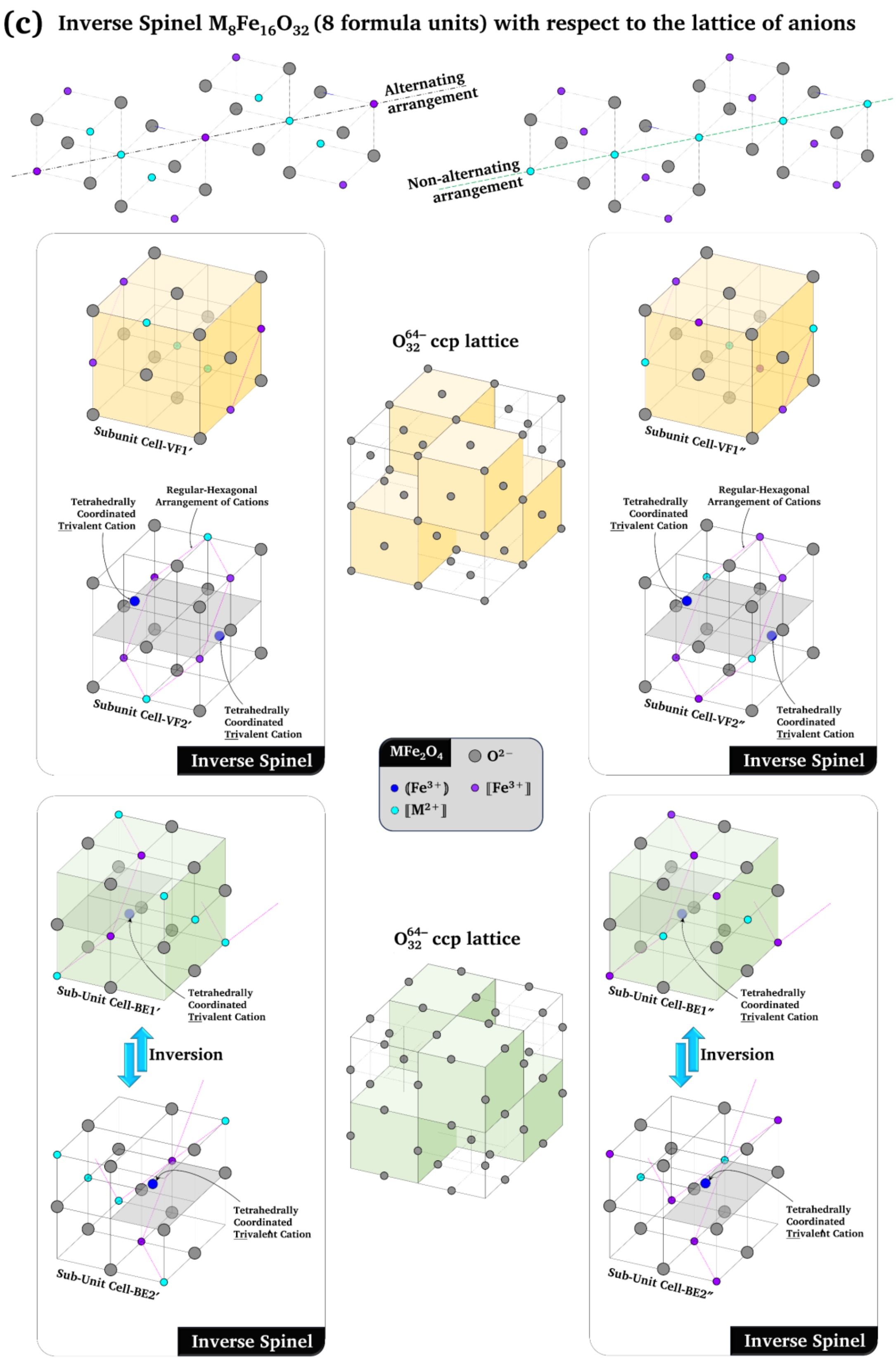

Fig. S1 *continued* (c) cubic unit cells of inverse spinel with respect to the ccp arrangement of the anions.

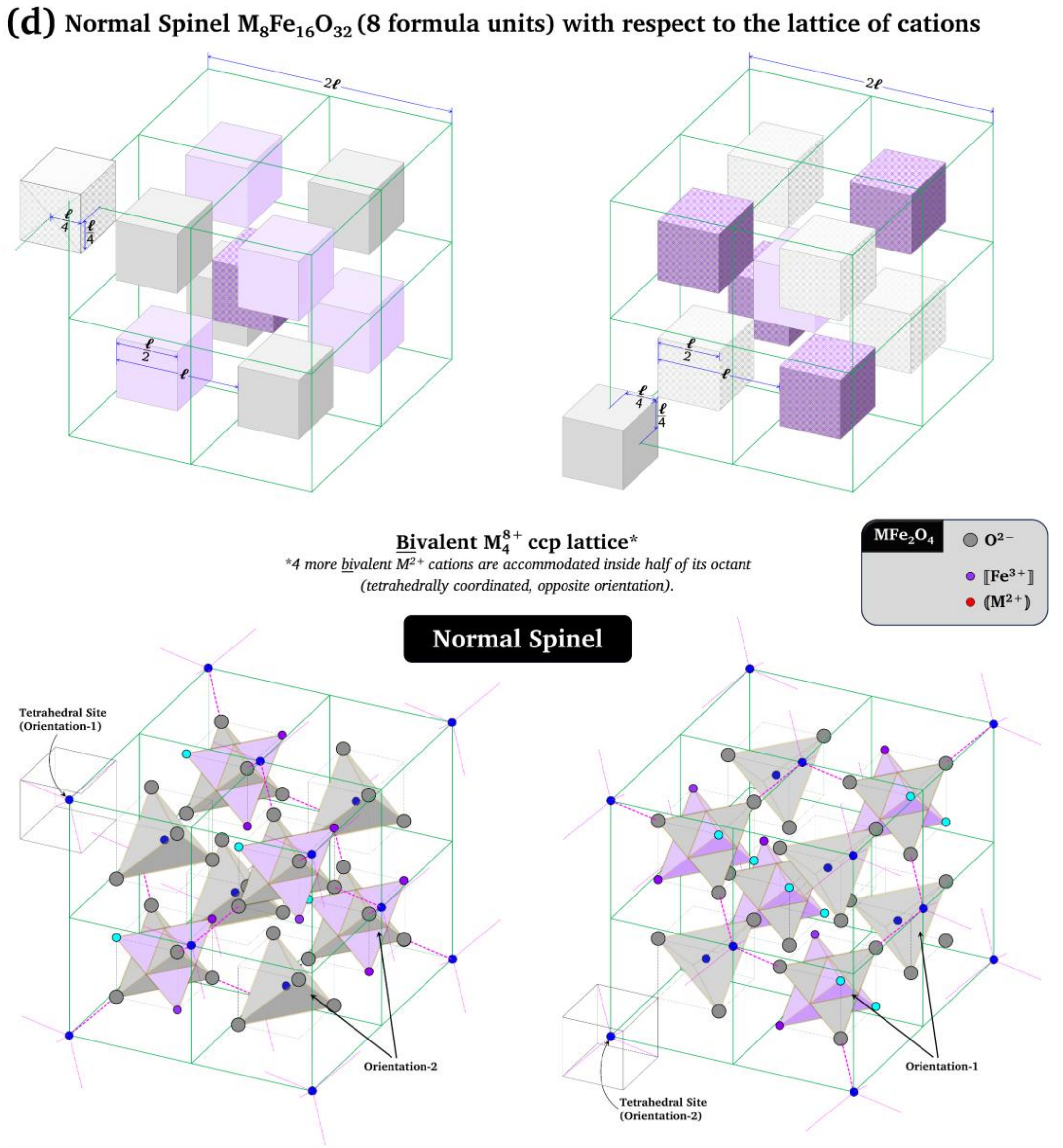

Fig. S1 *continued* (d) cubic unit cells of normal spinel with respect to the ccp arrangement of divalent cations.

(e) Inverse Spinel $M_8Fe_{16}O_{32}$ (8 formula units) with respect to the lattice of cations

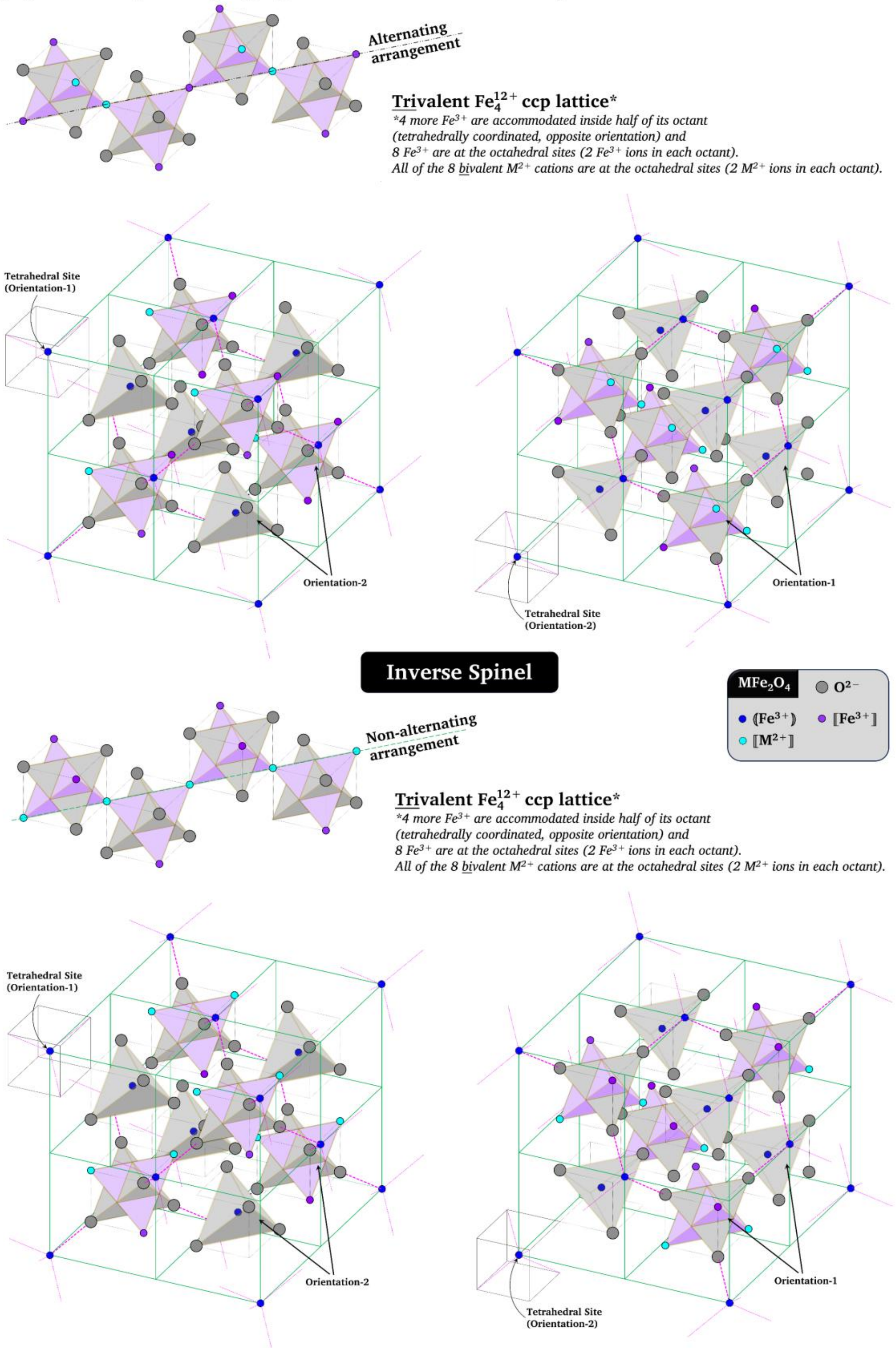

Fig. S1 *continued* (e) cubic unit cells of inverse spinel with respect to the ccp arrangement of trivalent cations.

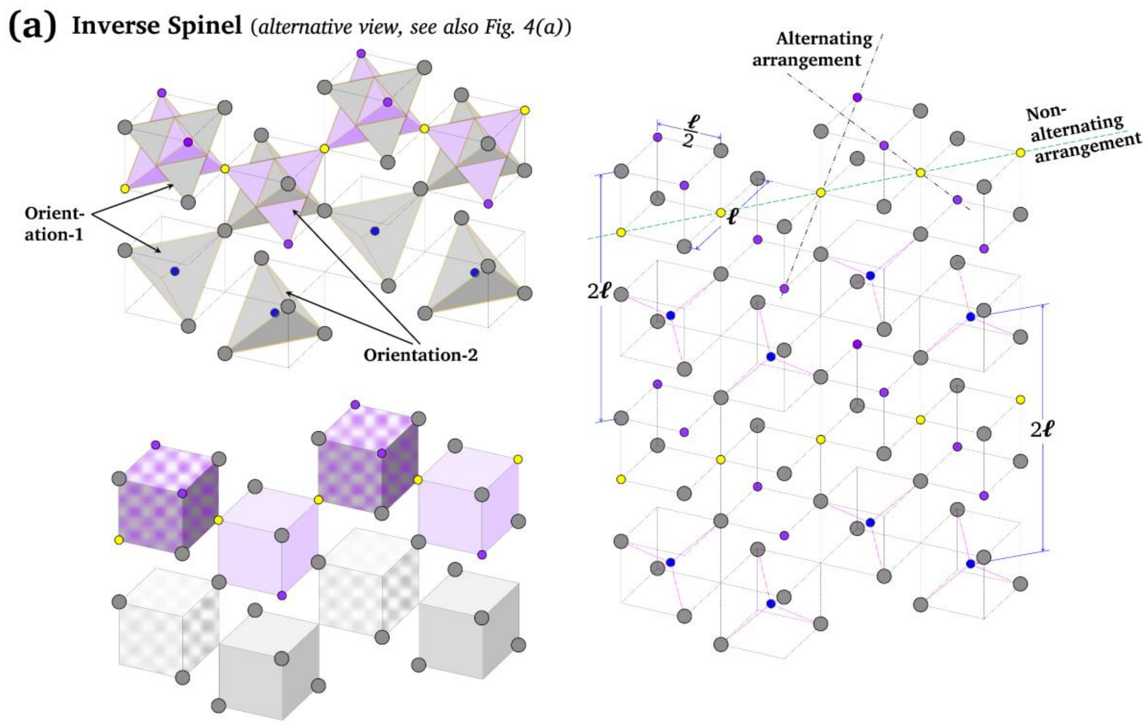

Fig. S2 (a) Alternative view of the inverse spinel structure.

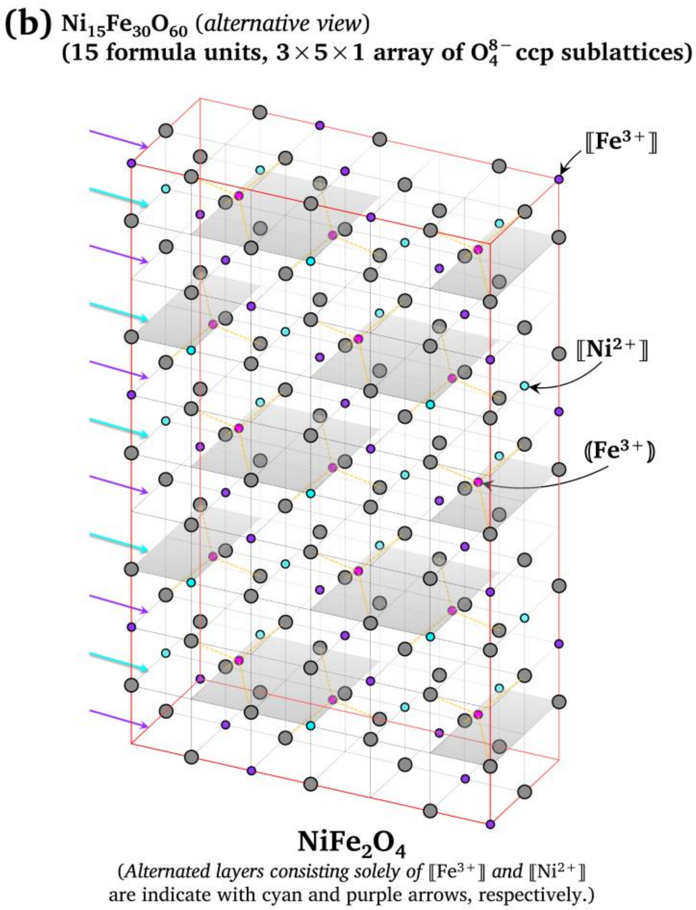

Fig. S2 *continued* (b) Alternative view of the structure of Ni ferrite based on the structure described in (a).
